# Assessment of potential risk factors associated with gestational diabetes mellitus: evidence from a Mendelian randomization study

**DOI:** 10.3389/fendo.2023.1276836

**Published:** 2024-01-08

**Authors:** Qingming Fu, Rumeng Chen, Shuling Xu, Yining Ding, Chunxia Huang, Binsheng He, Ting Jiang, Bin Zeng, Meihua Bao, Sen Li

**Affiliations:** ^1^ School of Stomatology, Changsha Medical University, Changsha, China; ^2^ School of Life Sciences, Beijing University of Chinese Medicine, Beijing, China; ^3^ The Hunan Provincial Key Laboratory of the TCM Agricultural Biogenomics, Changsha Medical University, Changsha, China; ^4^ Hunan key laboratory of the research and development of novel pharmaceutical preparations, School of Pharmaceutical Science, Changsha Medical University, Changsha, China

**Keywords:** risk factor, gestational diabetes mellitus, Mendelian randomization, UK biobank, FinnGen

## Abstract

**Background:**

Previous research on the association between risk factors and gestational diabetes mellitus (GDM) primarily comprises observational studies with inconclusive results. The objective of this study is to investigate the causal relationship between 108 traits and GDM by employing a two-sample Mendelian randomization (MR) analysis to identify potential risk factors of GDM.

**Methods:**

We conducted MR analyses to explore the relationships between traits and GDM. The genome-wide association studies (GWAS) for traits were primarily based on data from the UK Biobank (UKBB), while the GWAS for GDM utilized data from FinnGen. We employed a false discovery rate (FDR) of 5% to account for multiple comparisons.

**Results:**

The inverse-variance weighted (IVW) method indicated that the genetically predicted 24 risk factors were significantly associated with GDM, such as “Forced expiratory volume in 1-second (FEV1)” (OR=0.76; 95% CI: 0.63, 0.92), “Forced vital capacity (FVC)” (OR=0.74; 95% CI: 0.64, 0.87), “Usual walking pace” (OR=0.19; 95% CI: 0.09, 0.39), “Sex hormone-binding globulin (SHBG)” (OR=0.86; 95% CI: 0.78, 0.94). The sensitivity analyses with MR-Egger and weighted median methods indicated consistent results for most of the trats.

**Conclusion:**

Our study has uncovered a significant causal relationship between 24 risk factors and GDM. These results offer a new theoretical foundation for preventing or mitigating the risks associated with GDM.

## Introduction

Gestational diabetes mellitus (GDM) is the occurrence of hyperglycemia of varying severity due to impaired glucose tolerance, which is first diagnosed during pregnancy (1, 2). According to the International Diabetes Federation, it is estimated that GDM will affect one out of every six live newborns worldwide in 2019 (3). GDM significantly impacts both maternal and fetal health, as indicated by previous studies (4). Furthermore, GDM not only worsens short-term adverse outcomes during pregnancy (5–7) but also increases the long-term likelihood of developing type 2 diabetes mellitus (T2DM) among women (8, 9), which has been linked to various complications (10–16).

Observational research has identified multiple associations between various risk factors and GDM (17–19). However, these investigations are susceptible to confounding variables that may influence their findings. Additionally, the casual association proposed by these observational studies may lack statistical validity due to inconsistent study designs, conflicting findings, and substantial variability across different settings. Consequently, there is inadequate evidence available within these associations to establish a direct causal link between risk factors and GDM.

In order to address the aforementioned challenges, we employed Mendelian randomization (MR) as a method to mitigate biased estimation and reverse causation in the relationship between traits and GDM. In MR analysis, single nucleotide polymorphisms (SNPs), a type of genetic variation, are utilized as instrumental variables (IVs). Statistical techniques are utilized in this approach to evaluate the presence of a causal association between exposures and outcomes. Genetic variants serve as suitable IVs due to their random distribution during meiosis. Consequently, they exhibit reduced susceptibility to confounding influences. Hence, if these genetic variants are randomly distributed within a population, the observed causal relationships between exposures and outcomes are not likely due to potential confounders such as environmental risks, lifestyle choices, or socioeconomic status (20). Thus, MR design is employed in this study to systematically investigate the causal associations between 108 traits and GDM to identify the potential risk factors of GDM.

## Methods

### Study design

Utilizing datasets obtained from genome-wide association studies (GWAS), we identified significant SNPs associated with 108 traits as exposure variables. These SNPs were employed as IV, and a MR analysis was conducted to evaluate the causal relationship between the 108 traits and GDM.

### Data sources

To adhere to the principles of a two-sample MR design, we sourced exposure and outcome data from distinct European populations as previously described (21–23). We extracted minimally adjusted GWAS summary statistics for our variables of interest from the largest available sample. This dataset included individuals of both sexes and European or mixed ancestry. Our selection process for summary statistics of 108 exposure variables followed a previously described procedure outlined in **Supplementary Figure 1** (24). While most exposure GWAS studies utilized data from the UKBB (detailed information can be found in **Supplementary Table 1**), the dataset for GDM, as outcome, relied on information sourced from FinnGen, a significant biomedical research initiative based in Finland.

### Selection of IVs

IVs were selected for the MR analysis based on specific criteria. The criteria included a significant genetic relationship between IVs and exposure, with a *P*-value < 5× 10^-8^. Independent IVs were identified by performing clumping within a 10 Mb window and considering linkage disequilibrium (LD) with an R^2^ value below 0.001. Furthermore, following previous studies, only IVs with a minor allele frequency (MAF) greater than 0.01 were considered in our analysis. Palindromic SNPs were excluded from the analysis due to their intermediate allele frequencies (25). F-statistics were computed to assess the strength of the IVs; values exceeding 10 indicated reduced likelihood of weak instrument bias (refer to **Supplementary Table 2**) (26).

### MR analysis and sensitivity analysis

The main technique employed in the MR analysis was the IVW method. Furthermore, both the weighted median technique and MR-Egger approaches were utilized. The MR-Egger intercept test was employed to evaluate the existence of horizontal pleiotropy. To address potential outliers, pleiotropy-corrected data from MR-PRESSO were incorporated. The degree of heterogeneity was examined using the Cochrane Q value. We conducted a leave-one-out sensitivity analysis to evaluate how each IV impacted causal relationships and ensure robustness of findings. The calculation of causal effects involved the use of odds ratios (ORs) along with their corresponding 95% confidence intervals (CIs). Multiple comparisons were conducted using a false discovery rate (FDR) of 5%. All MR analyses in R were conducted using the TwoSampleMR package.

## Results

**Figure 1:**
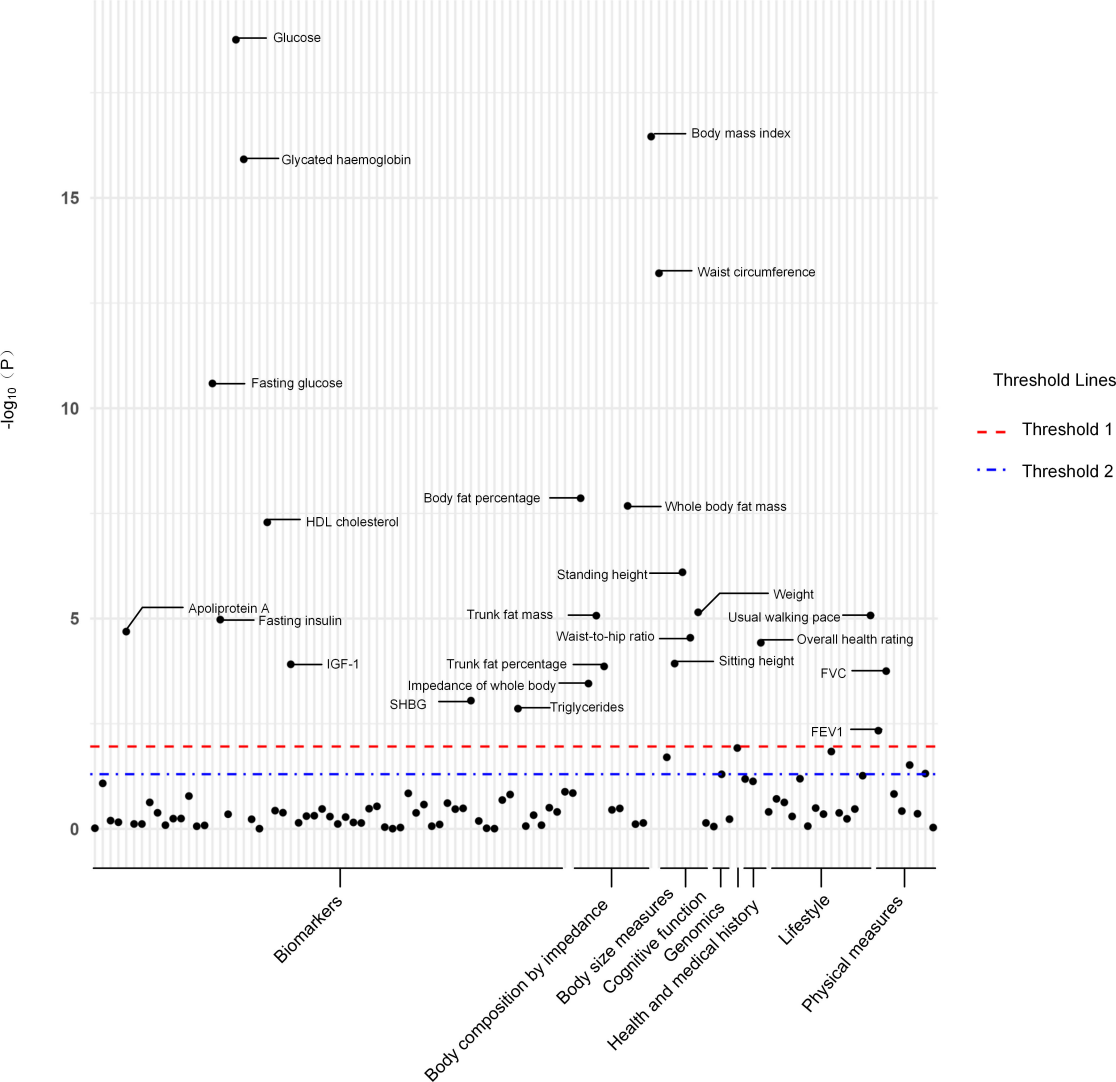
The *P*-value distribution of associations between 24 risk factors and GDM in the Mendelian randomization analysis. The red dashed line indicates the significance threshold adjusted by false discovery rate. The blue dash-dotted line indicates the suggestive significance threshold, set at *P* = 0.05. FVC, Forced vital capacity; FEV1, Forced expiratory volume in 1-second; HDL, High-density lipoprotein; IGF-1, Insulin-like growth factor 1; SHBG, Sex hormone-binding globulin.

**Figure 2:**
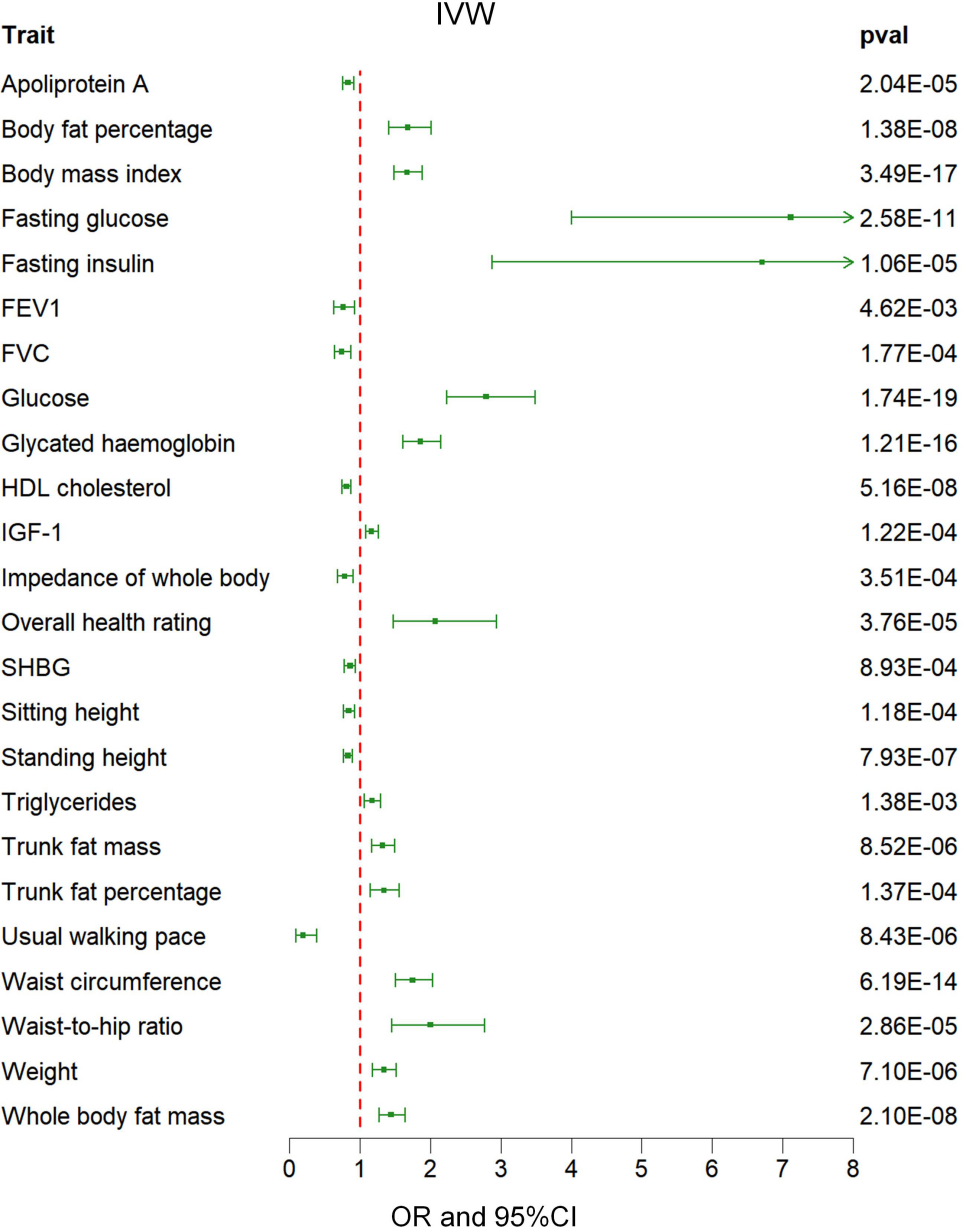
Associations between genetically predicted 24 risk factors and GDM examined by IVW methods. GDM, Gestational Diabetes Mellitus; IVW, inverse-variance weighted; FVC, Forced vital capacity; FEV1, Forced expiratory volume in 1-second; HDL, High-density lipoprotein; IGF-1, Insulin-like growth factor 1; SHBG, Sex hormone-binding globulin; OR, odds ratio; CI, confidence interval.

**Figure 3:**
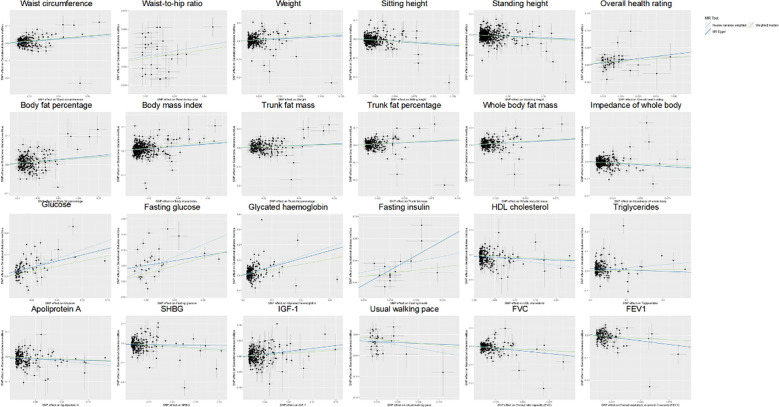
Scatter plot showing the causal effects of 24 risk factors on GDM. SNP, single nucleotide polymorphism; GDM, Gestational Diabetes Mellitus; HDL, High-density lipoprotein; SHBG, Sex hormone-binding globulin; IGF-1, Insulin-like growth factor 1; FVC, Forced vital capacity; FEV1, Forced expiratory volume in 1-second.

**Figure 4:**
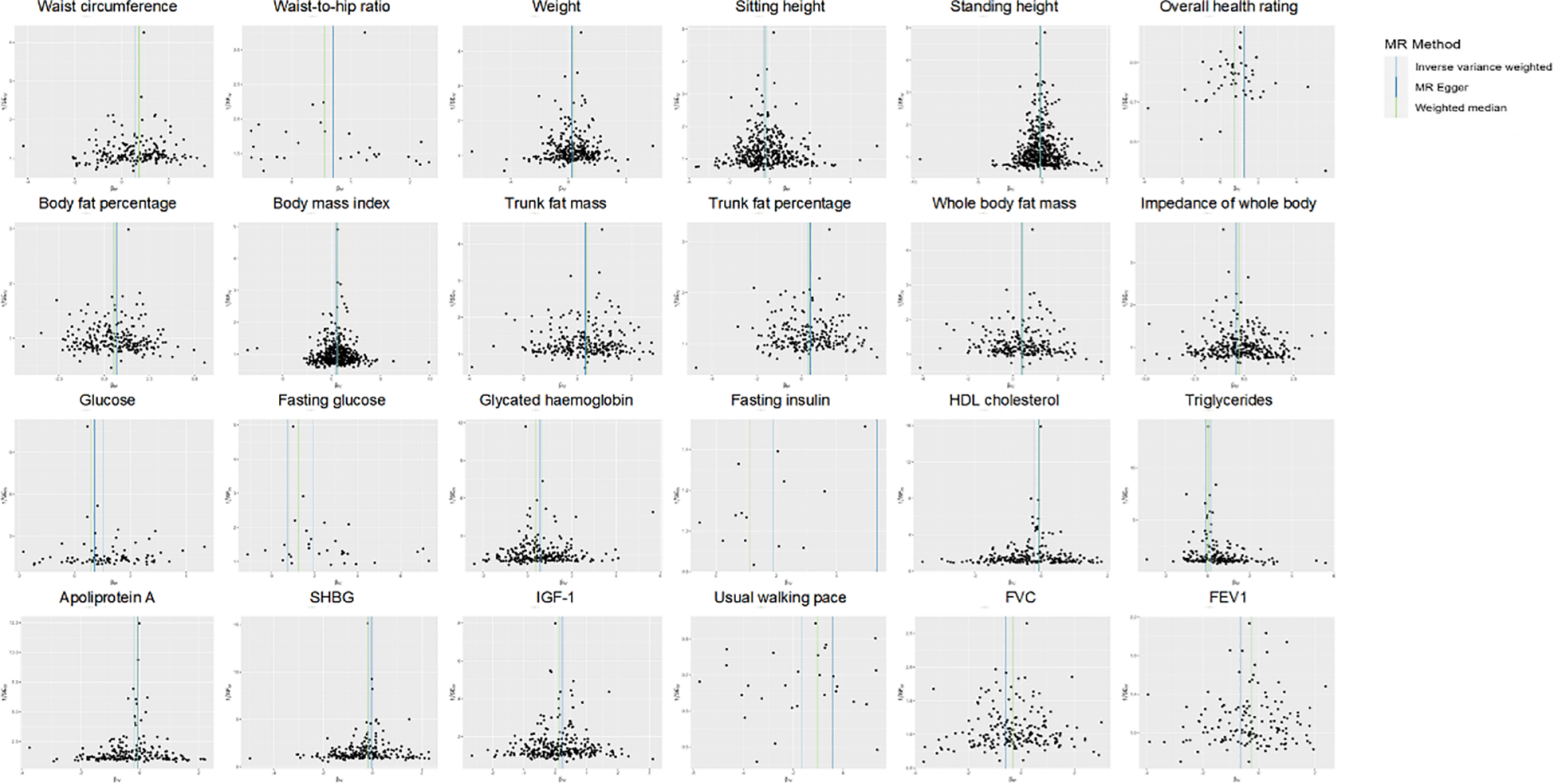
Funnel plot indicating the causal associations of 24 risk factors on GDM. SNP, single nucleotide polymorphism; GDM, Gestational Diabetes Mellitus; HDL, High-density lipoprotein; SHBG, Sex hormone-binding globulin; IGF-1, Insulin-like growth factor 1; FVC, Forced vital capacity; FEV1, Forced expiratory volume in 1-second; IV, instrumental variable; SE, standard error.

Out of a pool of 108 variables, SNPs were selected as IVs for potential risk factors according to predetermined inclusion and exclusion criteria. The findings were interpreted based on FDR-adjusted threshold. Using the IVW technique in MR analysis, we found significant relationships of 24 genetically predicted risk factors, such as “Apoliprotein A” (OR= 0.83; 95% CI: 0.76, 0.91), “Forced expiratory volume in 1-second (FEV1)” (OR=0.76; 95% CI: 0.63, 0.92), “Insulin-like growth factor 1 (IGF-1)” (OR=1.16; 95% CI: 1.08, 1.26) and “Usual walking pace” (OR=0.19; 95% CI: 0.09, 0.39), with GDM (**Figures 1**, **2**, **Supplementary Table 3**). The F-statistics for the IVs of the 24 risk factors ranged from 28.62 to 9445.10, indicating good instrument strength (**Supplementary Table 2**). Except for triglycerides, we found that 23 risk factors consistently showed a significant association with GDM in the same direction when analyzed using both MR-Egger and weighted median techniques (**Supplementary Table 3**). The scatter plot in **Figure 3** illustrated the causal relationships between all the 24 traits and GDM. The possible heterogeneity was also examined (**Figure 4**, **Supplementary Table 4**). Horizontal pleiotropy was estimated in our causality assessment based on analysis using MR-Egger technique as shown in **Supplementary Table 5**, and MR-PRESSO analyses indicated consistent findings after removing outlier IVs (**Supplementary Table 6**). The leave-one-out analysis demonstrated that no single SNP was solely responsible for the observed outcomes, as shown in **Supplementary Figure 2**.

## Discussion

This study employed a two-sample MR analysis to investigate the causal relationship between traits and GDM. The analysis incorporated GWAS summary statistics from public databases. The findings indicated significant causal associations between 24 risk factors and the risk of GDM. These risk factors will be discussed in detail across four subsequent paragraphs based on their respective categories.

### Body size measures and body composition by impedance analysis

Body size measurements and body composition evaluated using impedance analysis are important indicators for assessing obesity. Overweight or obese women have up to a four-fold increased risk of developing GDM (27). We found a significant causal relationship between GDM and various body size measurements such as body mass index (BMI), weight, waist-to-hip ratio (WHR), and waist circumference (WC) in our study. Additionally, we observed correlations among several measures of body composition determined through impedance analysis including trunk fat mass, trunk fat percentage, whole-body fat mass, and body-fat percentage. Previous studies have demonstrated a strong association between gestational weight gain and both gestational impaired glucose tolerance and GDM (28, 29). Obesity and being overweight are significant risk factors for acquiring GDM (4). A recent study revealed that obesity and visceral adiposity are correlated with an elevated risk of developing GDM. Furthermore, it highlighted that among these factors, visceral adiposity specifically poses a higher risk for GDM (17). BMI, as a measure of general obesity, has been reported to show an association with the prevalence of GDM. Specifically, there is evidence suggesting that every 1 kg/m² increase in pre-pregnancy BMI leads to a rise in GDM prevalence by 0.92% (30). Central obesity refers to an excessive accumulation of abdominal fat which can be assessed using markers such as WHR and WC measurements (31). Previous literature has demonstrated an association between maternal central obesity in the first trimester of pregnancy and a higher occurrence of GDM (31, 32). The presence of visceral adipose tissue can be easily explained as it directly contributes to the pathogenesis of hyperglycemia. It does so by secreting various substances such as thrombogenic agents, inflammatory compounds, and inhibitors of adiponectin. These substances negatively impact glucose metabolism, increase insulin resistance and facilitate the development of metabolic syndrome along with subsequent cardiovascular diseases (33, 34).

### Biomarkers

A meta-analysis revealed a significant association between elevated fasting glucose levels and a nearly two-fold increase in the risk of developing GDM (27). Moreover, a recent study has established an association between elevated fasting glucose during the initial stages of pregnancy and the subsequent onset of GDM (35). Enquobahrie et al. reported that for every increase in triglyceride content by 20 mg/dL, there is a 10% higher likelihood of developing GDM (36). Hypertriglyceridemia increases the risk for macrosomia due to factors such as insulin resistance caused by elevated triglycerides along with reduced lipoprotein lipase function. Macrosomia results in excessive fetal growth, obesity, as well as accumulation and release of fatty acids in cord blood and fetal adipose tissue (37). Previous research demonstrates an inverse correlation between serum HDL-c concentration and the risk of GDM and macrosomia. Additionally, even a slight increase in HDL-c levels serves as a protective factor against these conditions (38). Apolipoprotein A-1 is the primary lipoprotein associated with HDL-c. In contradiction to our results, a previous cohort study reported no association between serum Apolipoprotein A-1 levels, insulin resistance, or the risk of GDM in human subjects (39). However, since this study was observational, it cannot fully eliminate potential confounding variables as contributors to this discrepancy. Sex hormone binding globulin (SHBG), derived from the liver, is expressed in the placenta and acts as a regulator of sex steroid hormones. SHBG levels in the first trimester of pregnancy have been identified as reliable biomarkers for GDM (40, 41). There exists a negative correlation between SHBG and T2DM (42). Several previous studies have not only identified HbA1c as a diagnostic tool for GDM (43–45) but have also established a relationship between an HbA1c level above 7% in early pregnancy and adverse maternal outcomes (45). Fetal IGF-1 plays a crucial role in fetal growth due to its mitogenic and metabolic properties (46). According to Schwartz et al, an increase in IGF-1 concentration within umbilical cord blood contributes to accelerated intrauterine fetal growth (47).

### Physical measures

Forced vital capacity (FVC) and FEV1 are commonly used indicators of lung function (48, 49). Consistent with previous research, our study identified a significant inverse causal association between FVC, FEV1, and GDM. A prior study has reported a significant association between restrictive ventilatory dysfunction assessed through FVC and FEV1 measurements with an elevated risk of T2DM, whereas no such relationship was observed for obstructive ventilatory dysfunction evaluated using the FEV1/FVC ratio (50). Emerging evidence indicates that inflammatory markers such as C-reactive protein and interleukin-6 might contribute to the association between T2DM and decreased FEV1 and FVC (51).

### Lifestyle

Our study identified a negative causal relationship between usual walking pace and GDM, consistent with prior research in this field. Previous studies have suggested a robust correlation between the usual walking pace and a decreased likelihood of developing GDM (52). Furthermore, these studies revealed that women reporting faster speeds or longer durations during regular walks exhibited reduced risks of developing GDM when compared to those with slower speeds and shorter durations (52). Exercise leads to a significant increase in muscle glucose uptake. Exercise can increase muscle glucose uptake by up to 100 times when compared to resting conditions in humans (53). Increased glucose supply to the contracting skeletal muscles is made possible by the increase in blood flow and capillary recruitment during exercise (54).

### Strengths and limitations

The current study possesses three significant strengths. Firstly, previous observational studies have suggested an increased risk of GDM onset in association with long-term maternal residence in an environment characterized by a mixture of PM2.5, PM10, NO2, and PM2.5 chemical constituents (55). However, the MR method can help mitigate the influence of confounding factors on the results. This method involves selecting a SNP that is strongly associated with the exposure of interest as the IV. By utilizing this SNP, individuals can be randomly assigned to the exposure, ensuring comparability of the population in terms of both known and unknown confounders. Secondly, we conducted a comprehensive investigation of the causal relationship between 108 traits and GDM. Thirdly, we employed a variety of sensitivity analyses to verify the results of our main analyses using IVW method. Lastly, during our assessment of pleiotropy within the MR analysis framework, we utilized MR-PRESSO method to provide outlier-corrected results.

However, our study has several limitations. Firstly, the scope of our investigation was limited to people of European descent. Consequently, the generalizability is impacted by this restriction. Further studies on diverse population groups are still needed in future research. Secondly, there might be potential selection bias affecting our results as individuals who died due to competition risk related outcomes might be missed in the GWAS analysis. Thirdly, due to the utilization of GWAS summary data, we were unable to stratify the data by variables such as age and gender, potentially introducing population bias. Finally, we could not assess any potential non-linear association between risk factors and GDM. Future research utilizing extensive biobanks may offer insights into the complex relationship between traits and GDM.

## Conclusion

The present study has established a substantial and causative association between multiple risk factors and GDM. The MR analysis revealed statistically significant inverse associations of usual walking pace, FEV1 and FVC with GDM risk. This finding introduces novel insights that can guide future strategies aimed at preventing or mitigating the risks associated with GDM.

## Data availability statement

The original contributions presented in the study are included in the article/**Supplementary Material**. Further inquiries can be directed to the corresponding authors.

## Author contributions

QF: Writing – original draft. RC: Writing – original draft. SX: Writing – original draft. YD: Writing – original draft. CH: Writing – original draft. BH: Writing – original draft. TJ: Writing – original draft. BZ: Writing – original draft. MB: Writing – review & editing. SL: Writing – review & editing.
