# Supplementary material for: Assessment of potential risk factors associated with gestational diabetes mellitus: evidence from a Mendelian randomization study
Source: Front Endocrinol (Lausanne). 2024 Jan 8;14:1276836. doi: 10.3389/fendo.2023.1276836 (PMC10801737; doi:10.3389/fendo.2023.1276836)
Supplement: Supplementary file 1 [file DataSheet_1.docx]

**Supplementary Table 1**. Information of GWAS summary datasets used in MR analyses

| Trait | GWAS.ID | Population | Sex | Sample size |
| --- | --- | --- | --- | --- |
| Adiponectin | ieu-a-1 | Mixed | Males and Females | 39883 |
| Alanine aminotransferase | ukb-d-30620_irnt | European | Males and Females | 361194 |
| Albumin | ukb-d-30600_irnt | European | Males and Females | 361194 |
| Alcohol intake frequency. | ukb-a-25 | European | Males and Females | 336965 |
| Alcoholic drinks per week | ieu-b-73 | European | Males and Females | 335394 |
| Alkaline phosphatase | ukb-d-30610_irnt | European | Males and Females | 361194 |
| Apoliprotein A | ukb-d-30630_irnt | European | Males and Females | 361194 |
| Apoliprotein B | ukb-d-30640_irnt | European | Males and Females | 361194 |
| Aspartate aminotransferase | ukb-d-30650_irnt | European | Males and Females | 361194 |
| Basal metabolic rate | ukb-a-268 | European | Males and Females | 331307 |
| Basophill percentage | ukb-d-30220_irnt | European | Males and Females | 349861 |
| Birth weight | ukb-a-198 | European | Males and Females | 193063 |
| Birth weight of first child | ukb-a-318 | European | Males and Females | 145558 |
| Body fat | ieu-a-999 | European | Males and Females | 100716 |
| Body fat percentage | ukb-a-264 | European | Males and Females | 331117 |
| Body mass index | ieu-b-40 | European | Males and Females | 681275 |
| Calcium | ukb-d-30680_irnt | European | Males and Females | 361194 |
| Cholesterol | ukb-d-30690_irnt | European | Males and Females | 361194 |
| Cigarettes per Day | ieu-b-25 | European | Males and Females | 337334 |
| C-reactive protein | ukb-d-30710_irnt | European | Males and Females | 361194 |
| Creatinine | ukb-d-30700_irnt | European | Males and Females | 361194 |
| Creatinine (enzymatic) in urine | ukb-a-333 | European | Males and Females | 327525 |
| Current tobacco smoking | ukb-a-16 | European | Males and Females | 337030 |
| Cystatin C | ukb-d-30720_irnt | European | Males and Females | 361194 |
| Daytime dozing / sleeping (narcolepsy) | ukb-a-15 | European | Males and Females | 336082 |
| Diastolic blood pressure automated reading | ukb-a-359 | European | Males and Females | 317756 |
| Direct bilirubin | ukb-d-30660_irnt | European | Males and Females | 361194 |
| Drive faster than motorway speed limit | ukb-a-8 | European | Males and Females | 306030 |
| Eosinophill percentage | ukb-d-30210_irnt | European | Males and Females | 349861 |
| Fasting glucose | ieu-b-114 | European | Males and Females | 133010 |
| Fasting insulin | ieu-b-116 | European | Males and Females | 108557 |
| Fluid intelligence score | ukb-a-196 | European | Males and Females | 108818 |
| FEV1 | ukb-a-337 | European | Males and Females | 307638 |
| FVC | ukb-a-336 | European | Males and Females | 307638 |
| Gamma glutamyltransferase | ukb-d-30730_irnt | European | Males and Females | 361194 |
| Getting up in morning | ukb-a-10 | European | Males and Females | 336501 |
| Glucose | ukb-d-30740_irnt | European | Males and Females | 361194 |
| Glycated haemoglobin | ukb-d-30750_irnt | European | Males and Females | 361194 |
| Haematocrit percentage | ukb-d-30030_irnt | European | Males and Females | 350475 |
| Haemoglobin concentration | ukb-d-30020_irnt | European | Males and Females | 350474 |
| HDL cholesterol | ukb-d-30760_irnt | European | Males and Females | 361194 |
| Heart rate | ieu-a-1056 | Mixed | Males and Females | 92355 |
| Heel bone mineral density (BMD) T-score automated | ukb-a-500 | European | Males and Females | 194398 |
| High light scatter reticulocyte count | ukb-d-30300_irnt | European | Males and Females | 344729 |
| High light scatter reticulocyte percentage | ukb-d-30290_irnt | European | Males and Females | 344729 |
| Hip circumference | ukb-a-388 | European | Males and Females | 336601 |
| IGF-1 | ukb-d-30770_irnt | European | Males and Females | 361194 |
| Immature reticulocyte fraction | ukb-d-30280_irnt | European | Males and Females | 344728 |
| Impedance of whole body | ukb-a-269 | European | Males and Females | 331284 |
| LDL direct | ukb-d-30780_irnt | European | Males and Females | 361194 |
| Lipoprotein A | ukb-d-30790_irnt | European | Males and Females | 361194 |
| Lymphocyte count | ukb-d-30120_irnt | European | Males and Females | 349856 |
| Lymphocyte percentage | ukb-d-30180_irnt | European | Males and Females | 349861 |
| Mean corpuscular haemoglobin | ukb-d-30050_irnt | European | Males and Females | 350472 |
| Mean corpuscular haemoglobin concentration | ukb-d-30060_irnt | European | Males and Females | 350468 |
| Mean corpuscular volume | ukb-d-30040_irnt | European | Males and Females | 350473 |
| Mean platelet (thrombocyte) volume | ukb-d-30100_irnt | European | Males and Females | 350470 |
| Mean reticulocyte volume | ukb-d-30260_irnt | European | Males and Females | 344728 |
| Mean sphered cell volume | ukb-d-30270_irnt | European | Males and Females | 344729 |
| Mean time to correctly identify matches | ukb-a-199 | European | Males and Females | 335139 |
| Monocyte count | ukb-d-30130_irnt | European | Males and Females | 349856 |
| Monocyte percentage | ukb-d-30190_irnt | European | Males and Females | 349861 |
| Morning/evening person (chronotype) | ukb-a-11 | European | Males and Females | 301143 |
| Nap during day | ukb-a-12 | European | Males and Females | 337074 |
| Neuroticism score | ukb-a-230 | European | Males and Females | 274108 |
| Neutrophill count | ukb-d-30140_irnt | European | Males and Females | 349856 |
| Neutrophill percentage | ukb-d-30200_irnt | European | Males and Females | 349861 |
| Overall health rating | ukb-a-251 | European | Males and Females | 336020 |
| Past tobacco smoking | ukb-a-17 | European | Males and Females | 310749 |
| Peak expiratory flow (PEF) | ukb-a-338 | European | Males and Females | 307638 |
| Phosphate | ukb-d-30810_irnt | European | Males and Females | 361194 |
| Platelet count | ukb-d-30080_irnt | European | Males and Females | 350474 |
| Platelet crit | ukb-d-30090_irnt | European | Males and Females | 350471 |
| Platelet distribution width | ukb-d-30110_irnt | European | Males and Females | 350470 |
| Pulse rate automated reading | ukb-a-3 | European | Males and Females | 317756 |
| Red blood cell (erythrocyte) count | ukb-d-30010_irnt | European | Males and Females | 350475 |
| Red blood cell (erythrocyte) distribution width | ukb-d-30070_irnt | European | Males and Females | 350473 |
| Reticulocyte count | ukb-d-30250_irnt | European | Males and Females | 344729 |
| Reticulocyte percentage | ukb-d-30240_irnt | European | Males and Females | 344728 |
| SHBG | ukb-d-30830_irnt | European | Males and Females | 361194 |
| Sitting height | ukb-a-195 | European | Males and Females | 336172 |
| Sleep duration | ukb-a-9 | European | Males and Females | 335410 |
| Sleeplessness / insomnia | ukb-a-13 | European | Males and Females | 336965 |
| Sodium in urine | ukb-a-335 | European | Males and Females | 326831 |
| Standing height | ukb-a-389 | European | Males and Females | 336474 |
| Systolic blood pressure automated reading | ukb-a-360 | European | Males and Females | 317754 |
| telomere length | ieu-b-4879 | European | Males and Females | 472174 |
| Testosterone | ukb-d-30850_irnt | European | Males and Females | 361194 |
| Total bilirubin | ukb-d-30840_irnt | European | Males and Females | 361194 |
| Total cholesterol | ieu-a-301 | European | Males and Females | 187365 |
| Total protein | ukb-d-30860_irnt | Mixed | Males and Females | 361194 |
| Triglycerides | ukb-d-30870_irnt | European | Males and Females | 361194 |
| Trunk fat mass | ukb-a-291 | European | Males and Females | 331093 |
| Trunk fat percentage | ukb-a-290 | European | Males and Females | 331113 |
| Trunk fat-free mass | ukb-a-292 | European | Males and Females | 331030 |
| Trunk predicted mass | ukb-a-293 | European | Males and Females | 330995 |
| Urate | ukb-d-30880_irnt | European | Males and Females | 361194 |
| Urea | ukb-d-30670_irnt | European | Males and Females | 361194 |
| Urinary sodium-potassium ratio | ieu-b-72 | European | Males and Females | 326938 |
| Usual walking pace | ukb-a-513 | European | Males and Females | 335349 |
| Vitamin D | ukb-d-30890_irnt | European | Males and Females | 361194 |
| Waist circumference | ukb-a-382 | European | Males and Females | 336639 |
| Waist-to-hip ratio | ieu-a-72 | European | Males and Females | 224459 |
| Weight | ukb-a-249 | Mixed | Males and Females | 336227 |
| White blood cell (leukocyte) count | ukb-d-30000_irnt | European | Males and Females | 350470 |
| Whole body fat mass | ukb-a-265 | European | Males and Females | 330762 |
| Whole body fat-free mass | ukb-a-266 | European | Males and Females | 331291 |
| Whole body water mass | ukb-a-267 | European | Males and Females | 331315 |
| GDM | finn-GEST_DIABETES | European | Males and Females | 210870 |
| Abbreviation: GWAS: genome-wide association study; ID: Identification; MR: Mendelian randomization; UKB: UK Biobank; FEV1: Forced expiratory volume in 1-second; FVC: Forced vital capacity; HDL: High-density lipoprotein; IGF-1: Insulin-like growth factor 1; SHBG: Sex hormone-binding globulin; GDM: Gestational diabetes. | | | | |

**Supplementary Table 2**. The F-statistics of IVs

| GWAS.ID | Trait | 1. Statistic | | |
| --- | --- | --- | --- | --- |
|  |  | Min | | Max |
| UKB-d-30630_irnt | Apoliprotein A | 29.77 | 5502.15 | |
| UKB-a-264 | Body fat percentage | 30.01 | 433.59 | |
| ieu-b-40 | Body mass index | 28.62 | 1426.17 | |
| ieu-b-114 | Fasting glucose | 29.75 | 973.44 | |
| ieu-b-116 | Fasting insulin | 30.44 | 100 | |
| UKB-a-337 | FEV1 | 29.75 | 189.62 | |
| UKB-a-336 | FVC | 29.78 | 310.47 | |
| UKB-d-30740_irnt | Glucose | 29.80 | 3330.46 | |
| UKB-d-30750_irnt | Glycated haemoglobin | 29.94 | 8023.61 | |
| UKB-d-30760_irnt | HDL cholesterol | 29.81 | 9445.10 | |
| UKB-d-30770_irnt | IGF-1 | 29.72 | 2275.40 | |
| UKB-a-269 | Impedance of whole body | 29.79 | 702.43 | |
| UKB-a-251 | Overall health rating | 29.90 | 55.10 | |
| UKB-a-265 | Whole body fat mass | 29.75 | 636.20 | |
| UKB-d-30830_irnt | SHBG | 29.79 | 8219.67 | |
| UKB-a-195 | Sitting height | 29.88 | 1146.83 | |
| UKB-a-389 | Standing height | 29.73 | 1335.34 | |
| UKB-d-30870_irnt | Triglycerides | 29.76 | 5899.42 | |
| UKB-a-291 | Trunk fat mass | 29.79 | 546.41 | |
| UKB-a-290 | Trunk fat percentage | 29.76 | 354.65 | |
| UKB-a-513 | Usual walking pace | 30.02 | 84.47 | |
| UKB-a-382 | Waist circumference | 29.76 | 660.76 | |
| ieu-a-72 | Waist-to-hip ratio | 29.22 | 169.79 | |
| UKB-a-249 | Weight | 29.73 | 772.58 | |
| Abbreviation: IVs: instrumental variables; GWAS: genome-wide association study; ID: Identification; SNPs: single nucleotide polymorphisms; Min: Minimum; Max: Maximum; UKB: UK Biobank; FEV1: Forced expiratory volume in 1-second; FVC: Forced vital capacity; HDL: High-density lipoprotein; IGF-1: Insulin-like growth factor 1; SHBG: Sex hormone-binding globulin. | | | | |

**Supplementary Table 3**. MR analysis results

| Risk Factor | Method | OR | 95%CI | *P* |
| --- | --- | --- | --- | --- |
| Apoliprotein A | IVW | 0.83 | 0.76-0.91 | 2.04E-05 |
| Apoliprotein A | MR-Egger | 0.94 | 0.83-1.07 | 3.47E-01 |
| Apoliprotein A | WM | 0.92 | 0.82-1.02 | 1.27E-01 |
| Body fat percentage | IVW | 1.68 | 1.41-2.01 | 1.38E-08 |
| Body fat percentage | MR-Egger | 1.97 | 1.02-3.81 | 4.56E-02 |
| Body fat percentage | WM | 1.72 | 1.39-2.12 | 4.39E-07 |
| Body mass index | IVW | 1.67 | 1.48-1.88 | 3.49E-17 |
| Body mass index | MR-Egger | 1.61 | 1.18-2.2 | 3.17E-03 |
| Body mass index | WM | 1.87 | 1.59-2.2 | 2.43E-14 |
| Fasting glucose | IVW | 7.12 | 4-12.67 | 2.58E-11 |
| Fasting glucose | MR-Egger | 2.14 | 0.68-6.75 | 2.06E-01 |
| Fasting glucose | WM | 3.52 | 2.42-5.12 | 4.25E-11 |
| Fasting insulin | IVW | 6.71 | 2.88-15.65 | 1.06E-05 |
| Fasting insulin | MR-Egger | 211.25 | 3.45-12921.24 | 2.54E-02 |
| Fasting insulin | WM | 3.10 | 1.48-6.46 | 2.59E-03 |
| FEV1 | IVW | 0.76 | 0.63-0.92 | 4.62E-03 |
| FEV1 | MR-Egger | 0.53 | 0.28-0.99 | 4.73E-02 |
| FEV1 | WM | 0.76 | 0.61-0.95 | 1.79E-02 |
| FVC | IVW | 0.74 | 0.64-0.87 | 1.77E-04 |
| FVC | MR-Egger | 0.55 | 0.35-0.86 | 9.85E-03 |
| FVC | WM | 0.73 | 0.6-0.9 | 2.64E-03 |
| Glucose | IVW | 2.79 | 2.23-3.49 | 1.74E-19 |
| Glucose | MR-Egger | 2.06 | 1.44-2.93 | 1.45E-04 |
| Glucose | WM | 1.81 | 1.51-2.17 | 1.88E-10 |
| Glycated haemoglobin | IVW | 1.86 | 1.61-2.15 | 1.21E-16 |
| Glycated haemoglobin | MR-Egger | 1.69 | 1.32-2.17 | 4.43E-05 |
| Glycated haemoglobin | WM | 1.43 | 1.24-1.64 | 4.54E-07 |
| HDL cholesterol | IVW | 0.81 | 0.75-0.87 | 5.16E-08 |
| HDL cholesterol | MR-Egger | 0.93 | 0.83-1.04 | 1.95E-01 |
| HDL cholesterol | WM | 0.92 | 0.83-1.02 | 1.15E-01 |
| IGF-1 | IVW | 1.16 | 1.08-1.26 | 1.22E-04 |
| IGF-1 | MR-Egger | 1.26 | 1.08-1.47 | 3.02E-03 |
| IGF-1 | WM | 1.13 | 1-1.27 | 5.77E-02 |
| Impedance of whole body | IVW | 0.78 | 0.68-0.9 | 3.51E-04 |
| Impedance of whole body | MR-Egger | 0.67 | 0.46-0.97 | 3.57E-02 |
| Impedance of whole body | WM | 0.77 | 0.65-0.91 | 2.38E-03 |
| Overall health rating | IVW | 2.07 | 1.47-2.94 | 3.76E-05 |
| Overall health rating | MR-Egger | 3.50 | 0.44-27.73 | 2.41E-01 |
| Overall health rating | WM | 2.06 | 1.27-3.34 | 3.58E-03 |
| SHBG | IVW | 0.86 | 0.78-0.94 | 8.93E-04 |
| SHBG | MR-Egger | 0.98 | 0.85-1.12 | 7.36E-01 |
| SHBG | WM | 0.83 | 0.75-0.92 | 4.68E-04 |
| Sitting height | IVW | 0.84 | 0.77-0.92 | 1.18E-04 |
| Sitting height | MR-Egger | 0.75 | 0.6-0.94 | 1.28E-02 |
| Sitting height | WM | 0.79 | 0.71-0.9 | 1.55E-04 |
| Standing height | IVW | 0.83 | 0.77-0.89 | 7.93E-07 |
| Standing height | MR-Egger | 0.87 | 0.74-1.02 | 8.59E-02 |
| Standing height | WM | 0.82 | 0.74-0.9 | 9.07E-05 |
| Triglycerides | IVW | 1.17 | 1.06-1.29 | 1.38E-03 |
| Triglycerides | MR-Egger | 0.94 | 0.82-1.07 | 3.45E-01 |
| Triglycerides | WM | 1.04 | 0.93-1.16 | 5.18E-01 |
| Trunk fat mass | IVW | 1.32 | 1.17-1.49 | 8.52E-06 |
| Trunk fat mass | MR-Egger | 1.35 | 0.93-1.97 | 1.16E-01 |
| Trunk fat mass | WM | 1.43 | 1.23-1.66 | 3.08E-06 |
| Trunk fat percentage | IVW | 1.34 | 1.15-1.56 | 1.37E-04 |
| Trunk fat percentage | MR-Egger | 1.47 | 0.85-2.55 | 1.71E-01 |
| Trunk fat percentage | WM | 1.38 | 1.14-1.65 | 7.34E-04 |
| Usual walking pace | IVW | 0.19 | 0.09-0.39 | 8.43E-06 |
| Usual walking pace | MR-Egger | 0.67 | 0.01-58.79 | 8.61E-01 |
| Usual walking pace | WM | 0.36 | 0.16-0.82 | 1.56E-02 |
| Waist circumference | IVW | 1.75 | 1.51-2.03 | 6.19E-14 |
| Waist circumference | MR-Egger | 2.08 | 1.32-3.28 | 1.84E-03 |
| Waist circumference | WM | 2.08 | 1.72-2.52 | 8.05E-14 |
| Waist-to-hip ratio | IVW | 2.00 | 1.45-2.77 | 2.86E-05 |
| Waist-to-hip ratio | MR-Egger | 2.00 | 0.45-8.94 | 3.71E-01 |
| Waist-to-hip ratio | WM | 1.74 | 1.2-2.52 | 3.61E-03 |
| Weight | IVW | 1.34 | 1.18-1.52 | 7.10E-06 |
| Weight | MR-Egger | 1.25 | 0.89-1.75 | 1.95E-01 |
| Weight | WM | 1.45 | 1.24-1.69 | 2.99E-06 |
| Whole body fat mass | IVW | 1.44 | 1.27-1.64 | 2.10E-08 |
| Whole body fat mass | MR-Egger | 1.50 | 1.01-2.22 | 4.38E-02 |
| Whole body fat mass | WM | 1.57 | 1.35-1.83 | 6.43E-09 |
| Abbreviation: MR: Mendelian randomization; OR: odds ratio; CI: confidence interval; SNP: single nucleotide polymorphism; IVW: inverse-variance weighted; WM: weighted median; FEV1: Forced expiratory volume in 1-second; FVC: Forced vital capacity; HDL: High-density lipoprotein; IGF-1: Insulin-like growth factor 1; SHBG: Sex hormone-binding globulin. | | | | |

**Supplementary Table** **4**. Heterogeneity test results

| Risk factor | Q_*P* |
| --- | --- |
| Apoliprotein A | 4.66E-11 |
| Body fat percentage | 7.78E-21 |
| Body mass index | 1.18E-38 |
| Fasting glucose | 2.43E-31 |
| Fasting insulin | 4.41E-05 |
| FEV1 | 1.12E-07 |
| FVC | 7.47E-07 |
| Glucose | 3.22E-45 |
| Glycated haemoglobin | 3.11E-158 |
| HDL cholesterol | 2.31E-09 |
| IGF-1 | 5.05E-06 |
| Impedance of whole body | 9.42E-20 |
| Overall health rating | 4.33E-01 |
| SHBG | 1.68E-25 |
| Sitting height | 1.74E-12 |
| Standing height | 4.90E-15 |
| Triglycerides | 8.24E-27 |
| Trunk fat mass | 1.57E-20 |
| Trunk fat percentage | 1.67E-17 |
| Usual walking pace | 6.63E-03 |
| Waist circumference | 2.13E-11 |
| Waist-to-hip ratio | 9.77E-05 |
| Weight | 4.98E-30 |
| Whole body fat mass | 9.77E-23 |
| Abbreviation: FEV1: Forced expiratory volume in 1-second; FVC: Forced vital capacity; HDL: High-density lipoprotein; IGF-1: Insulin-like growth factor 1; SHBG: Sex hormone-binding globulin. | |

**Supplementary Table 5**. MR-Egger pleiotropy test results

| Risk factor | Egger_intercept | *SE* | *P* |
| --- | --- | --- | --- |
| Apoliprotein A | -5.64E-03 | 2.27E-03 | 1.40E-02 |
| Body fat percentage | -2.49E-03 | 5.14E-03 | 6.28E-01 |
| body mass index | 6.18E-04 | 2.60E-03 | 8.12E-01 |
| Fasting glucose | 3.59E-02 | 1.55E-02 | 2.78E-02 |
| Fasting insulin | -5.78E-02 | 3.45E-02 | 1.20E-01 |
| FEV1 | 6.80E-03 | 5.64E-03 | 2.30E-01 |
| FVC | 5.57E-03 | 3.97E-03 | 1.63E-01 |
| Glucose | 1.29E-02 | 6.02E-03 | 3.56E-02 |
| Glycated haemoglobin | 3.75E-03 | 4.03E-03 | 3.54E-01 |
| HDL cholesterol | -6.68E-03 | 1.98E-03 | 8.81E-04 |
| IGF-1 | -2.56E-03 | 2.15E-03 | 2.35E-01 |
| Impedance of whole body | 2.87E-03 | 3.20E-03 | 3.71E-01 |
| Overall health rating | -6.44E-03 | 1.28E-02 | 6.17E-01 |
| SHBG | -6.20E-03 | 2.51E-03 | 1.42E-02 |
| Sitting height | 2.50E-03 | 2.39E-03 | 2.97E-01 |
| Standing height | -1.08E-03 | 1.80E-03 | 5.47E-01 |
| Triglycerides | 1.15E-02 | 2.57E-03 | 1.26E-05 |
| Trunk fat mass | -5.48E-04 | 4.03E-03 | 8.92E-01 |
| Trunk fat percentage | -1.76E-03 | 5.22E-03 | 7.36E-01 |
| Usual walking pace | -1.29E-02 | 2.31E-02 | 5.81E-01 |
| Waist circumference | -3.29E-03 | 4.23E-03 | 4.37E-01 |
| Waist-to-hip ratio | -1.54E-05 | 1.87E-02 | 9.99E-01 |
| Weight | 1.43E-03 | 3.32E-03 | 6.67E-01 |
| Whole body fat mass | -8.10E-04 | 4.04E-03 | 8.41E-01 |
| Abbreviation: *SE*: standard error; *P*:*P*-value; FEV1: Forced expiratory volume in 1-second; FVC: Forced vital capacity; HDL: High-density lipoprotein; IGF-1: Insulin-like growth factor 1; SHBG: Sex hormone-binding globulin. | | | |

**Supplementary Table 6**. MR-PRESSO analyses between exposures and outcomes with identified outliers

| Exposure | MR Analysis | Causal Estimate | SD | *P*-value |
| --- | --- | --- | --- | --- |
| Body mass index | Outlier-corrected | 0.554 | 0.052 | 1.03E-23 |
| Fasting glucose | Outlier-corrected | 1.921 | 0.215 | 6.08E-09 |
| Fasting insulin | Outlier-corrected | 1.452 | 0.317 | 6.34E-04 |
| Sitting height | Outlier-corrected | -0.201 | 0.041 | 1.79E-06 |
| Weight | Outlier-corrected | 0.331 | 0.057 | 1.80E-08 |
| Body fat percentage | Outlier-corrected | 0.601 | 0.085 | 1.77E-11 |
| Whole body fat mass | Outlier-corrected | 0.437 | 0.060 | 5.40E-12 |
| Impedance of whole body | Outlier-corrected | -0.238 | 0.062 | 1.62E-04 |
| Trunk fat percentage | Outlier-corrected | 0.345 | 0.075 | 6.55E-06 |
| Trunk fat mass | Outlier-corrected | 0.338 | 0.059 | 2.21E-08 |
| FVC | Outlier-corrected | -0.314 | 0.074 | 3.08E-05 |
| FEV1 | Outlier-corrected | -0.264 | 0.089 | 3.51E-03 |
| Waist circumference | Outlier-corrected | 0.588 | 0.071 | 1.58E-14 |
| Standing height | Outlier-corrected | -0.170 | 0.036 | 2.24E-06 |
| Apoliprotein A | Outlier-corrected | -0.169 | 0.040 | 3.81E-05 |
| Glucose | Outlier-corrected | 1.100 | 0.099 | 9.70E-18 |
| Glycated haemoglobin | Outlier-corrected | 0.516 | 0.053 | 3.13E-19 |
| HDL cholesterol | Outlier-corrected | -0.260 | 0.042 | 3.90E-09 |
| IGF-1 | Outlier-corrected | 0.118 | 0.038 | 1.87E-03 |
| SHBG | Outlier-corrected | -0.173 | 0.040 | 2.68E-05 |
| Triglycerides | Outlier-corrected | 0.193 | 0.041 | 6.58E-06 |
| Abbreviation: MR: Mendelian randomization; SD: standard deviation; FEV1: Forced expiratory volume in 1-second; FVC: Forced vital capacity; HDL: High-density lipoprotein; IGF-1: Insulin-like growth factor 1; SHBG: Sex hormone-binding globulin. | | | | |


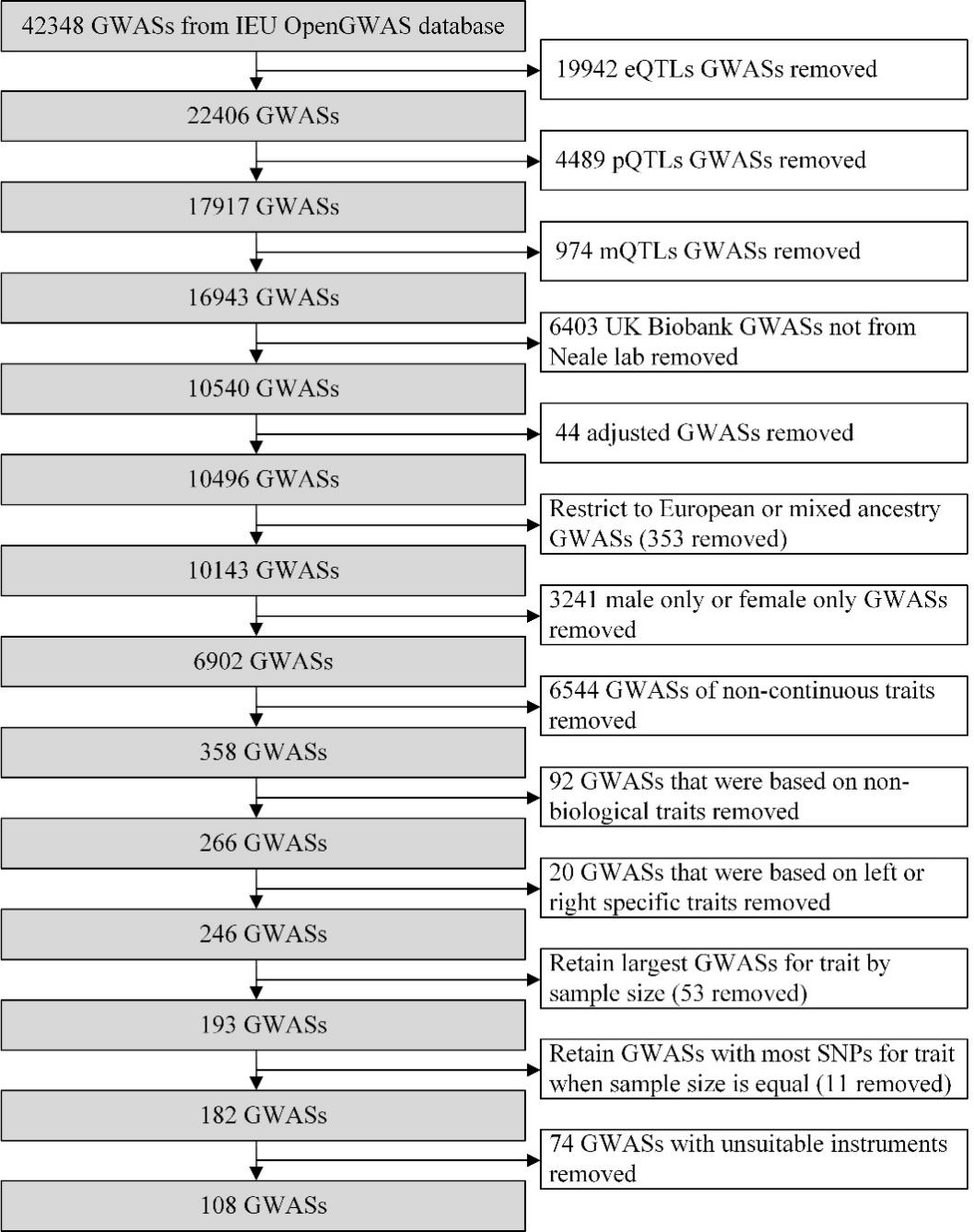


**Supplementary Figure 1**. Flowchart showing the trait selection procedure for causal analyses. GWAS: genome-wide association study.


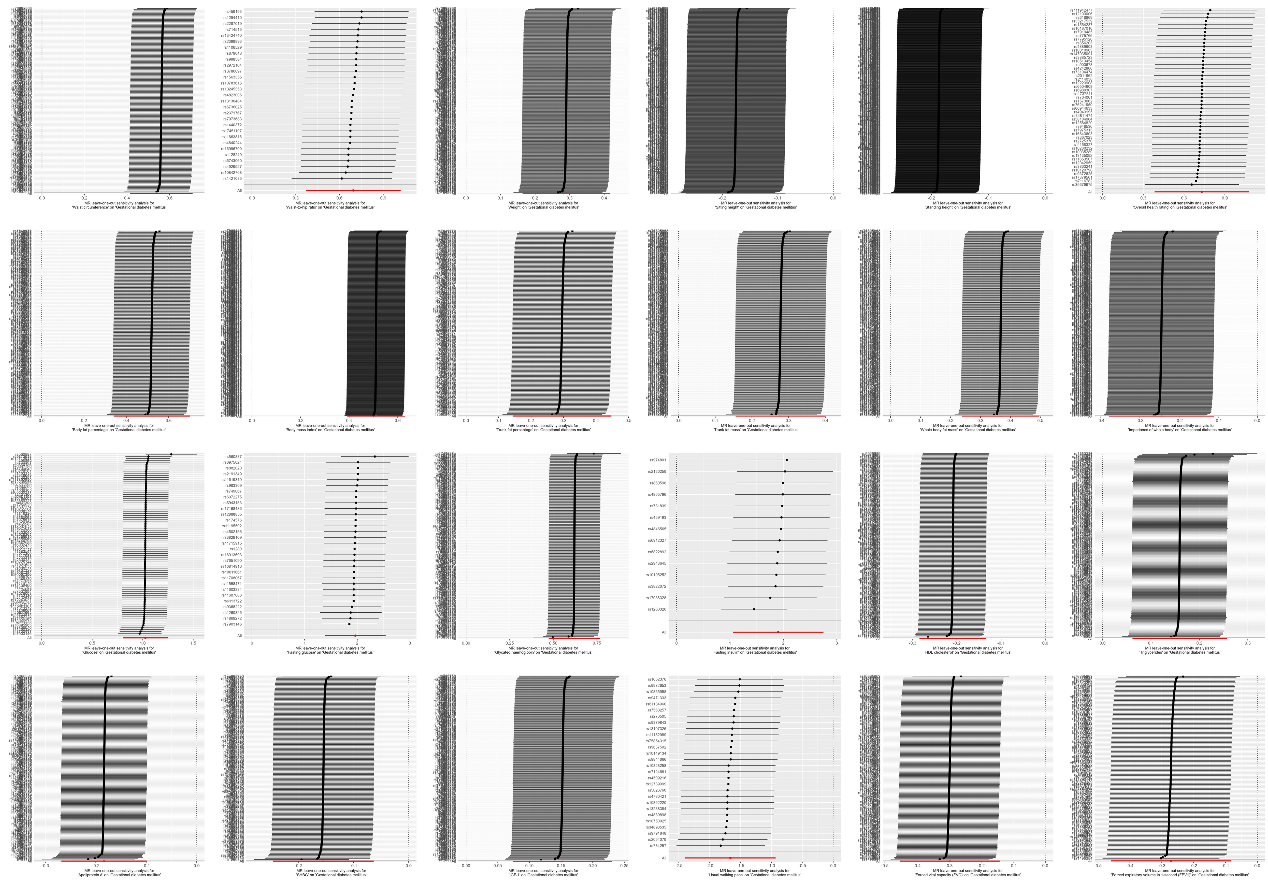


**Supplementary Figure 2**. Leave-one-out sensitivity analysis examining the causal estimates of 24 risk factors on GDM by the IVW method after exclude a specific SNP from the analysis. The red line represents the IVW estimate of all SNPs on each outcome. MR: Mendelian randomization; SNP: single nucleotide polymorphism; GDM: Gestational Diabetes Mellitus; HDL: High-density lipoprotein; SHBG: Sex hormone-binding globulin; IGF-1: Insulin-like growth factor 1; IVW: inverse-variance weighted.
